# A Survey on Reinforcement Learning for Reconfigurable Intelligent Surfaces in Wireless Communications

**DOI:** 10.3390/s23052554

**Published:** 2023-02-24

**Authors:** Annisa Anggun Puspitasari, Byung Moo Lee

**Affiliations:** Department of Intelligent Mechatronics Engineering and Convergence Engineering for Intelligent Drone, Sejong University, Seoul 05006, Republic of Korea

**Keywords:** intelligent reflecting surface (IRS), optimization, passive reflections, reconfigurable intelligent surface (RIS), reinforcement learning (RL), wireless communication

## Abstract

A reconfigurable intelligent surface (RIS) is a development of conventional relay technology that can send a signal by reflecting the signal received from a transmitter to a receiver without additional power. RISs are a promising technology for future wireless communication due to their improvement of the quality of the received signal, energy efficiency, and power allocation. In addition, machine learning (ML) is widely used in many technologies because it can create machines that mimic human mindsets with mathematical algorithms without requiring direct human assistance. Meanwhile, it is necessary to implement a subfield of ML, reinforcement learning (RL), to automatically allow a machine to make decisions based on real-time conditions. However, few studies have provided comprehensive information related to RL algorithms—especially deep RL (DRL)—for RIS technology. Therefore, in this study, we provide an overview of RISs and an explanation of the operations and implementations of RL algorithms for optimizing the parameters of RIS technology. Optimizing the parameters of RISs can offer several benefits for communication systems, such as the maximization of the sum rate, user power allocation, and energy efficiency or the minimization of the information age. Finally, we highlight several issues to consider in implementing RL algorithms for RIS technology in wireless communications in the future and provide possible solutions.

## 1. Introduction

**Figure 1:**
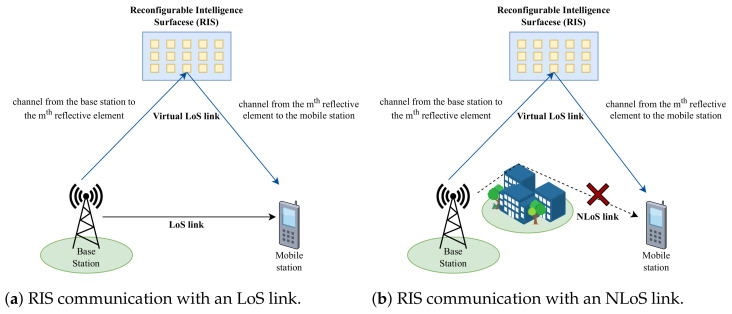
Illustration of RIS communication in wireless communication.

Reconfigurable intelligent surfaces (RISs) are some of the most promising emerging technologies for wireless communication networks in the future because they are able to provide several advantages, such as ease of development, low cost, and increased spectral and power efficiencies [1]. An RIS forwards a signal from a source node to a destination node by reflecting electromagnetic waves (EMs). Therefore, the destination node receives signals composed of elements from a direct line-of-sight (LoS) link and a reflective link, as shown in Figure 1a. However, the destination can still receive signals from the virtual LoS path, even when only a non-line-of-sight (NLoS) link is available, as illustrated in Figure 1b. Thus, this helps to increase the received signal quality and reduce interference [2].

**Figure 2:**
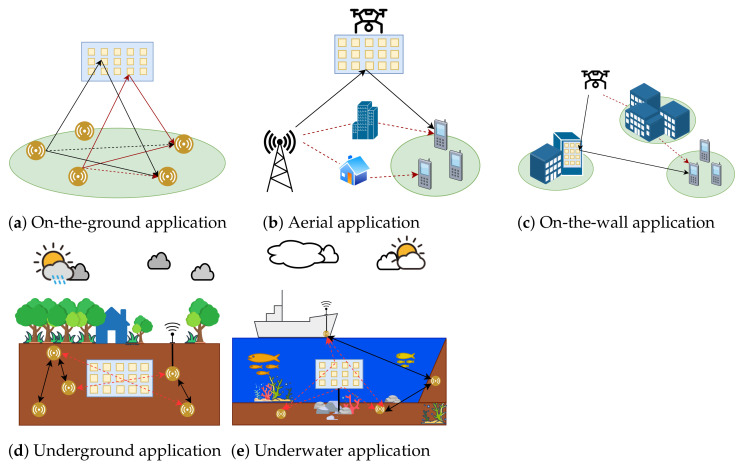
Application fields of RISs.

Using an artificial planar surface allows an RIS to control communications and is a break from cooperative communication systems with conventional relay technology. In [3], the proposed RIS could overcome the problem of power allocation among ground users in non-orthogonal multiple-access (NOMA) networks. Similarly, other authors proposed an RIS as a relay that improved the throughput performance of the backscatter link system among ground users in [4]. A capacity maximization was carried out in [5], where an intelligent reflecting surface (IRS) was used to optimize the IRS phase-shift coefficient and the transmit covariance matrices by distributing signals among multiple transmitters and receivers. However, in [6], the authors proposed an aerial RIS to enhance the power allocation and network coverage in micro-wave channels. Similarly, in [7], beamforming and RIS phase-shift optimization were achieved by implementing an aerial RIS-assisted NOMA network. In addition to hovering in the air while being carried by unmanned aerial vehicles (UAVs), RISs can be attached to the walls of a room or building. In [8], an RIS-assisted fingerprint base method was proposed, where an RIS was placed on a wall for indoor multi-user localization. In [9,10,11], an RIS was attached to a building to pass on communications among a mobile UAV, a fixed-position UAV, and ground users. In addition, an RIS can also be applied underground or underwater. In a recent study, the authors proposed the use of an RIS at the entrances of underground parking lots to increase the value of the signal-to-noise ratio (SNR) for communication between a base station (BS) placed outside the parking area and users inside the underground parking lot [12]. In [13,14], an RIS was implemented to enhance the outage performance and increase the channel capacity by assisting the mixed communication of radio frequency (RF) and underwater wireless optical communication (UWOC) systems. Thus, RIS technology is a passive component that can be used in a wide range of indoor or outdoor applications [15], as illustrated in Figure 2. Therefore, a controllable phase shift toward the receiver is essential for getting better performance. An optimal phase shift in an RIS is capable of maximizing the total number of served devices, the SNR value, and the network sum rate [2], mitigating signal propagation impairments [16], and increasing the covered area and the energy collection capacity in a hybrid scenario [17].

Technological progress has proceeded linearly with the rapid development of advances in communication technology that we have recently seen in academia and industry. Therefore, the demand for technology that is flexible and adaptive to environmental changes is also increasing. Machine learning (ML), which is a branch of artificial intelligence (AI), is a sophisticated technological breakthrough that continues to rapidly develop in various technological sectors, including wireless communication networks. One of the ML types, reinforcement learning (RL), is a way to overcome concerns about adaptive environmental changes due to its efficacy in embedded optimization and algorithms for instant decision making in wireless networks. Different RL algorithms have been adapted for RIS-based wireless communication to improve the performance. The categories of RL implemented in RISs are categorized as the deep Q-network (DQN), deep deterministic policy gradient (DDPG), twin delayed DDPG (TD3), and proximal policy optimization (PPO). Previous studies of RL implementation showed significant results when it was applied to RIS-assisted wireless communication systems [18], RIS-assisted UAV networks [19], and RIS-assisted NOMA [20]. RL provides an advantage for wireless communication because it allows the system to learn and build knowledge about radio channels without knowing the channel model and mobility pattern. The algorithm automatically observes the rewards from the environment to find solutions to the required optimization problems. Due to this way of working, the implementation of RL in an RIS system can provide advantages, as described in the studies mentioned earlier.

### 1.1. Related Work

Due to the promising advantages of implementing RISs for wireless communication in the future, recently, there have been several surveys discussing the application of RISs [21,22,23,24,25,26,27]. In [21], Kisseleff et al. focused on the implementation of RISs in underwater, underground, industrial, and disaster environments. They carried out a performance analysis of RIS deployment and system design in challenging environments. They highlighted that RISs could enhance the SINR performance by overcoming the problem of signal scattering/reflection caused by multipath effects and partially compensating for signal absorption through passive beamforming. Issues raised by the authors included uneven surfaces, water flows, and the movement of maritime objects in underwater media, types of soil conductivity with varying degrees of absorption in underground media, blocked sensors and mobile infrastructure in industrial environments, and broken infrastructure in a disaster environment. The authors of [22] focused on explaining the role of RIS hardware and system design technology. In addition, the authors also presented an explanation regarding the several implementation structures of RISs and implementations using electrical control technologies. Another study highlighted various applications of IRSs for assisting UAV communication networks and emerging technologies that could empower the advantages of RISs by focusing on ground and airborne scenarios [23]. The authors briefly stated that machine learning and deep learning are among the emerging technologies for improving channel estimation, embedded optimization, spectral efficiency, and other trade-offs. The authors of [24] discussed improvements in spectral efficiency, energy efficiency, security, and other types of performance as the effects of IRS position and the roles of UAVs for non-terrestrial networks by analyzing some performance criteria. This survey divided its analysis into different RIS positions and UAV roles in five scenarios.

In addition, due to the development of ML implementations, most of the existing literature has focused on implementations thereof for RISs [25,26,27]. An analysis regarding the optimization of UAV position and trajectory, RIS phase shift, and precoding at the base station was studied in [25]. The authors also mentioned several ML techniques that were implemented to perform the optimizations. Another study explained the operating principle of RISs and channel estimation in RIS technology. Their survey presented the model architectures of ML and its application in channel estimation, spectrum sensing, RIS phase shift, security, and energy efficiency in wireless networks [26]. In [27], Li et al. presented an analysis of optimization and AI methods while considering the solution quality and computational complexity. The authors focused on reviewing the RIS phase-shift optimization from the point of view of signal processing and AI. They divided their explanation into three variations depending on the different amplitude values for the RISs’ reflecting elements (REs).

Even though there have been several studies discussing the application of ML in RISs, they discussed general knowledge of RL and future opportunities for its implementation. However, there are still limited surveys that have dug into the performance of several RL algorithms in RIS technologies in depth. Table 1 summarizes the existing literature on the implementation of RLs for RISs in wireless communications.

### 1.2. Scope and Contributions

An adaptive system that can adapt to the current environmental conditions due to the rapid pace of environmental change and development can be obtained by implementing RL in an RIS. Compared to recent surveys regarding the implementation of ML in RISs, this study focuses on reviewing the role of RL algorithms that have been implemented for RISs in various emerging technologies. Due to the limited number of surveys that were specifically based on RL applications in RISs, this work fills the gap by providing information regarding RL algorithms for the optimization of RIS technology to overcome various wireless communication problems. The main contributions of our study are listed as follows:

- In the beginning, we give brief insights into RIS technology and RL. We provide a comprehensive introduction to RIS technology, including the types of RISs in terms of reflector types and phase-shift coefficient values.
- We provide a mathematical explanation of the RL algorithms presented in the literature. We categorize the different RL algorithms as DQN, DDPG, TD3, and PPO. We conduct a comprehensive review of the peculiarities, including the implementation of each RL algorithm in RIS technologies mentioned.
- We carry out an extensive analysis of the role of RL in empowering the use of this RIS integration by optimizing several parameters to solve various types of problems in several emerging technologies and application scenarios. The problems found in RIS technologies that can be solved by implementing RL algorithms are described as the energy efficiency, spectral efficiency, network capacity, security, and age of information.
- In the end, we discuss several existing and potential challenges while providing possible solutions as future research opportunities for overcoming the issues and honing the research work dedicated to this promising integration of RISs and RL algorithms.

### 1.3. Organization of the Paper

**Figure 3:**
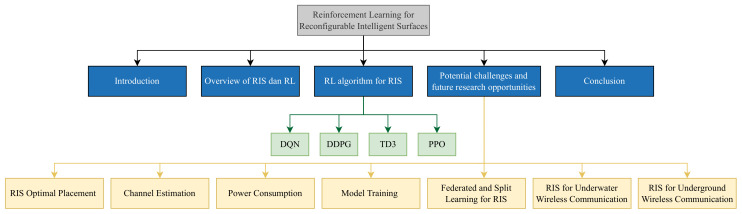
Organization of the paper.

As presented in Figure 3, the rest of this paper is organized as follows. An overview of RISs and RL is discussed in Section 2. Section 3 provides the RL algorithms implemented for RIS technology in the literature. Furthermore, we offer the potential challenges and future research opportunities in Section 4. Finally, Section 5 presents the conclusions of the paper.

## 2. An Overview of RISs and RL

### 2.1. RIS Technology

RISs are a development of conventional relay technology, and they forward radio-frequency signals from a transmitter to a receiver by reflecting them. An RIS is a two-dimensional array of meta-surface scatterers connected to a controller so that the variables can be changed and adjusted. The advantage of an RIS over its predecessor is that, because it consists of many REs that can simultaneously transmit different signals, an RIS can increase the spectrum efficiency [28]. In addition, the energy efficiency is also increased due to the active or passive reflection of signals [29].

#### 2.1.1. Active and Passive RISs

**Figure 4:**
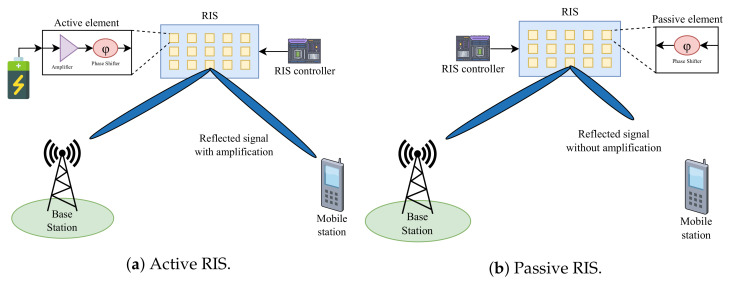
Comparison of active and passive RISs.

Functionally, both types of RISs have the same function of reflecting EMs. However, in the active reflector, each RE has an active load impedance to reflect the signal and amplify its electromagnetic power level [30]. The active load can be obtained by utilizing the negative resistance to convert the direct current (DC) signal into an RF signal. Amplifiers can be placed on each RE or several REs in a sub-array served by the same amplifier with different phase-shift circuits [31]. Compared to active RISs, passive RISs are usually more affordable and energy efficient because they do not require additional power to amplify the reflected signal. However, it takes a massive number of REs to avoid path loss [32,33]. Another way to overcome this issue is by adding a control system to the passive RIS, which makes phase shift essential when reflecting the received signal. Figure 4 shows a comparison between active and passive RISs.

#### 2.1.2. Continuous and Discrete RISs

Each RE in an RIS has a phase and amplitude setting that is made by a controller to adjust its electromagnetic response, which is a phase shift. The desired phase shift can be adjusted via the on or off status of the multiple pin diodes that are connected [34,35]. Phase-shift control can increase the spectrum and energy efficiency of an RIS due to the reflection of the signal without adding a power amplifier [36]. The discrete phase-shift coefficient is practically limited to discrete values between 0 and 2π with the same space. Meanwhile, the continuous RIS phase shift can take any values within the same range [37,38].

In [39], active and passive RISs with a continuous phase shift were implemented to analyze the achievable rate and power budget. The achievable rate for the active RIS was 55% higher than that for the passive RIS given the same number of RIS REs, with the cost of a higher hardware complexity. In addition, the passive RIS showed better performance for small power budgets. However, in [40], both active and passive IRSs were implemented with a continuous phase shift to measure the energy efficiency values in multi-user communication. The average energy efficiency value was monotonically increased with the number of IRS elements for both active and passive IRSs. However, for the passive IRS, the energy efficiency was increased by up to 5.75% compared with systems with an equal power-splitting active IRS. In [41], a hybrid passive–active RIS with a continuous phase shift was applied to maximize the minimum rate of multi-users. As the transmission power budget increased, the minimum rate of the passive RIS remained consistent because it did not require additional power. The minimum rate obtained by the active RIS at a low transmit power budget was worse than that of the passive RIS. Still, it increased up to 75% as the maximum transmit power increased at the RIS, with the cost of power consumption also increasing. However, by implementing a hybrid active–passive RIS, the minimum rate obtained with a low transmit power budget was higher than that of the active and passive RISs. As the transmit power budget increased, it grew up to 65% with respect to that of the passive RIS.

In [42], a passive RIS was implemented with continuous and discrete phase shifts. The performance of the RIS scheme increased with increasing RIS REs and transmit power and with either a continuous or a discrete phase shift. However, the continuous-phase-shift RIS produced an average sum rate that was 4.25% better than that of the discrete-phase-shift RIS. However, in [43], joint active–passive beamforming for IRS-assisted secure communication was implemented with both types of IRS phase shifts. The IRS with a continuous phase shift produced a higher sum secrecy rate than that of the IRS with a discrete phase shift. However, as the quantization order of the IRS phase shift rose, the sum secrecy rate significantly increased and gradually approached the performance of the algorithm of the IRS with a continuous phase shift. Both phase shifts were also implemented in [44] to configure the energy and spectral efficiencies of an active RIS in MIMO uplink transmission. Continuous phase shift—whether with partial or perfect CSI—showed a higher spectral efficiency than that of the discrete phase shift with one- or two-bit quantization. However, the substantially higher static-hardware-dissipated power of the continuous phase shift negatively influenced the energy efficiency. By implementing a discrete phase shift, the energy efficiency increased by up to 77.78% with respect to the continuous one.

Based on the description above, continuous and discrete phase-shift control benefits RIS implementation with the respective tradeoffs for both active and passive RISs. There are various ways of optimizing the RIS phase shift. Some studies have optimized the RIS phase shift by applying conventional optimization algorithms [45,46,47], machine learning, deep learning, and reinforcement learning [48,49,50].

### 2.2. RL Algorithm

**Figure 5:**
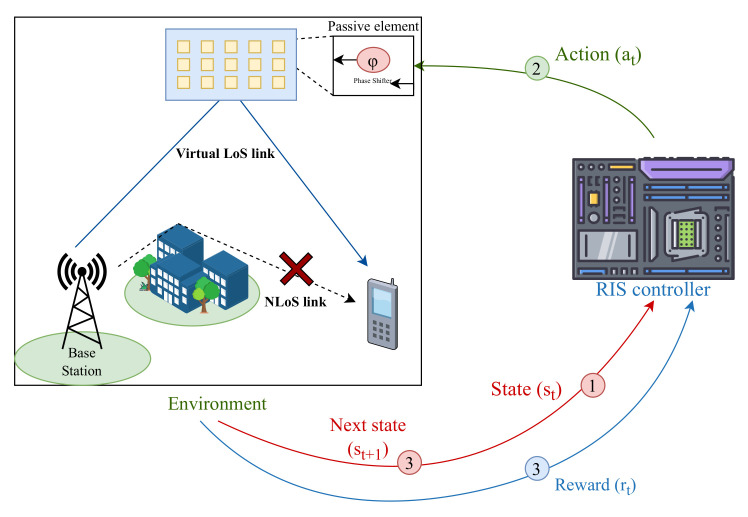
Reinforcement learning model.

Due to its ability to allow machines to automatically make decisions based on an analysis of their dataset collection, machine learning has been a breakthrough in the world of technology since its emergence. RL is a development of machine learning—deep learning, to be precise. In the RL algorithm, a machine interacts with a dynamic environment [51,52]. Thus, the algorithm learns from its own experience, which is stored in a dataset, to obtain the best decision results [53]. Mathematically based on the Markov decision process (MDP) formula, the RL algorithm contains three stages—state, action, and reward—as illustrated in Figure 5.

- State: a collection of the environment’s characteristics (*S*) sent by the environment to the agent. The input is the initial state $s_{1}$, and $s_{t}\in S$ denotes the environment at the time step *t*.
- Action: a collection of actions that are the response of the agent (*A*) to the received environmental characteristics. Every time the agent gives the action $a_{t}\in A$ at time instant *t*, the environment will send the agent the latest environment characteristics or what is called the next state $s_{t+1}$.
- Reward: a collection of feedback from the environment to the action sent by the agent (*R*). For every $r_{t}$ at time instant *t*, the environment will reward the agent when the results obtained are better than the results that were previously achieved. On the other hand, the environment will carry out a punishment when the results obtained are worse than before.
- Q-value function: a state–action value function that measures the cumulative reward value received by agent Q(s,a). Q-value indicates how good the action $a_{t}$ taken for the given state $s_{t}$ was.

Based on the method of deciding which action to take, there are two types of RL: policy-based and value-based RL methods [54]. A value-based method selects an action by considering the optimal value of the Q-value function $Q^{*}\left(s,a\right)$, while a policy-based method considers the optimal policy value or transition probability $\pi^{*}\left(s,a\right)$.

Combining deep neural networks (DNNs) with RL provides the added advantage of allowing the solution of extremely complex problems. In a value-based DRL method, the DNN acts as a function approximator that estimates the Q-value Q(s,a), as in (1).

(1) Q(s,a;θ)≈Q(s,a)

where θ is the weight of the DNN as a function parameter. However, in a policy-based RL method, the DNN functions as a gradient estimator ${▿}_{\theta }J\left(\theta \right)$ to estimate the probability value of J(θ), as shown in (2).

(2) $${▿}_{\theta }J\left(\theta \right)\approx \sum_{t\geq 0}r\left(\tau \right){▿}_{\theta }\log\pi_{\theta }\left(a_{t},st\right)$$

where r(τ) is the reward for each trajectory (path), and $\log\pi_{\theta }\left(a_{t},st\right)$ is the probability of the action taken for each state.

This study will specifically discuss two value-based RL methods (DQN and DDQN) and three policy-based RL methods (DDPG, TD3, and PPO) applied to an RIS technology. In general, value-based RL methods have a simpler architecture, so the time consumed is lower than that of policy-based RL methods. However, they have the characteristics of a discrete action space, which does not correspond much with most variables in an actual situation. Along with its development, recently, there have been studies implementing DRL in various branches of technology. Some existing studies have analyzed performance comparisons between value-based and policy-based methods. In [55], DRL methods were implemented for residential heating, ventilation, and air conditioning. Because the output size of the value-based RL method was larger than that of the policy-based one, the average return gained by the value-based RL method was 33.33% lower than that of the policy-based RL method. However, in [56], both RL methods were implemented for energy management in data-driven driving scenarios; the energy management strategy with the value-based method had a 0.06444% degradation in battery capacity compared to the 0.07178% degradation obtained with the policy-based method over the whole test cycle. Another study implemented a policy-based DRL method for controller development that allowed robust flying UAVs in a dynamic environment and compared it with a value-based method [57]. The policy-based method reached a converged state of 1000 episodes faster than the value-based method did, with a 33.3% higher average reward; it successfully hit the target 6.3% more often. Both of the DRL methods can also be implemented for energy consumption prediction. In [58], both methods were used to predict power consumption, and the policy-based method produced better system performance for a computation time that was 45.8% longer. The mean absolute error, root mean square error, and mean absolute percentage error values were 25.52%, 9.1%, and 21.13% smaller than those of the value-based method.

The following section will provide a comparison of both RL methods, as well as qualitative and quantitative analyses of the application of these RL methods in RIS-assisted wireless communication.

## 3. RL Algorithm for RISs

The DRL algorithm can be implemented in RIS technology for several things, especially optimization for phase shift, passive/active beamforming, resource allocation, power allocation, etc.

### 3.1. Deep Q-Network (DQN)

DQN is a value-based RL algorithm that seeks its best action based on the highest Q-value with the critical parameters considered in the Q-learning. It will iterate the Q-value for each observation to find the maximum value, as shown in (3) and (4).

(3) $$Q\left(s,a\right)\leftarrow Q\left(s,a\right)+\alpha Q^{*}\left(s,a\right)$$

(4) $$Q\left(s,a\right)\leftarrow Q\left(s,a\right)+\alpha \left\{r+\gamma \underset{a^{\prime }}{max}Q^{*}\left(s^{\prime },a^{\prime }\right)-Q\left(s,a\right)\right\}$$

where the maximum value of Q-value Q(s,a) is considered as the best action. Several studies have implemented the DQN algorithm for RIS technologies. Table 2 provides a brief summary of the studies that are presented below.

In [59], Wang et al. focused on the trajectory optimization of an IRS-assisted UAV communication system. This research implemented two DRL algorithms: DQN and DDPG. The results showed that DQN had a low computational complexity compared to that of the other implemented DRL algorithms due to its simpler structure. This was proven in this study, as the training time required by DQN was 1000–2000 seconds faster as the number of REs increased. However, it should be noted that the system design when using the DQN algorithm could only optimize the UAV trajectory in a limited discrete action space. Therefore, the DDPG achieved a better UAV trajectory because it always tried continuous actions, so the possibility of achieving a better quality of communication was higher. Even so, the level of energy efficiency of the system with the DQN implementation was much higher than that in the other two scenarios with random and fixed UAV movements.

Some other studies implemented advancements in DQN by using double DQN (DDQN). DDQN means that two NNs are used; the first DQN trains the original network, and the other handles the target network. The authors of [60] implemented DDQN to optimize the 3D-trajectory and phase-shift design for RIS-assisted UAV systems. DDQN was used here to model the UAV trajectory, flight time, and ground terminal (GT) scheduling as the discrete action. Partially solving the problems mentioned here by using traditional methods would cause enormous computation costs to be incurred. Thus, Mei et al. proposed a DRL-based solution in order to address those problems. The achieved results illustrated that the propulsion energy needed was 18.12% more efficient, thus increasing energy efficiency by up to 12.28% with respect to the system implementation without an RIS or optimal passive RIS phase shift. However, when the UAV trajectory and flight time were considered continuous variables, the discrete actions became enormous at the cost of a loss of accuracy due to the formulation of those continuous variables as discrete ones. Therefore, the authors leveraged the DDPG algorithm to find the optimal continuous value, which will be discussed in the following subsection.

In the other case, another study implemented DDQN in a model-free IRS configuration in a complex smart radio environment [61]. In this work, the current reflection pattern’s incremental phase shift was considered as an action. The DDQN algorithm in this study increased the total capacity by up to 42.86% for various Rician factor values compared to the other benchmarks. However, the action space was restrained for a fast convergence rate in DRL, which limited the phase freedom of the IRS. Thus, to obtain this signal quality, the authors added another model-free real-time optimization method to design the fine phase control of the RIS at the cost of increased time computation, as the dither-based method needed to sample the channels. Thus, the time resources dedicated to this method should be deliberately selected according to the channel block dynamics to balance the allocation of time between channel estimation and data transmission.

However, a characteristic of DQN, its discrete action space, can be a limitation when compared to reality, where everything is sustainable. If we want to implement continuous action by using DQN, getting an infinite number of actions will take forever, considering that DQN requires one output neuron per action. In addition, based on (3), it is difficult to determine the best action based on the maximum Q-value because DQN trains the given state simultaneously with all available actions. Thus, it is difficult to calculate the maximum value of the Q-value in the next state $Q^{*}$ with the DQN update rule. One way that can be used as a solution for overcoming these limitations is training a side estimator to get the best action before training it with the given state.

### 3.2. Deep Deterministic Policy Gradient (DDPG)

DDPG is a policy-based RL method and an actor–critic algorithm. There are two parameters in the DDPG algorithm: policy parameters—as actors—are denoted by $\pi_{\theta }\left(a,s\right)$, and critical parameters—as critics—are denoted by $Q_{\phi }^{{}_{\pi_{\theta }}}\left(s,a\right)$. DDPG trains actors to estimate the best action. After that, they train the action with the given state to know how good the action is for the state. All updates in DDPG are based on scholastic gradient descent (SGD) by using an adaptive gradient descent technique, such as resilient propagation (RProp), root mean square propagation (RMSprop), an adaptive gradient (Adagrad), Adam, etc. DDPG uses the Q-learning algorithm to find the optimal policy value $\pi^{*}$, as shown in (5).

(5) $$Q^{*}\left(s^{\prime },a^{\prime }\right)=\gamma Q_{\phi }^{{}_{\pi_{\theta }}}\left(s^{\prime },\pi^{\prime }\right)-Q_{\phi }^{{}_{\pi_{\theta }}}\left(s,a\right)$$

The equation above shows that DDPG does not consider the action, but rather the policy, which is the action provided by the actor. The DDPG algorithm trains the actor by using a deterministic policy gradient over the action, which is the input of the network, as shown in (6), and the target value for each sample i is shown in (7).

(6) $$\frac{J\theta }{\partial x}=\frac{1}{N)}\sum_{i}^{}\frac{Q_{\phi }^{{}_{\pi_{\theta }}}\left(s,a\right)}{da}\frac{\pi (s|\theta )}{d\theta }$$

(7) $$y_{i}=r_{i}+\gamma \underset{a^{\prime }}{max}{Q^{\prime }}_{\phi }^{\pi_{\theta }}\left(s^{\prime },a|\phi^{\prime }\right)$$

In the previous subsection, we discussed the characteristics of DQN technology for RISs. In the following studies, we will provide a brief summary of the papers discussed in this subsection, which are summarized in Table 3.

As mentioned in the previous subsection, the authors of [59] added the DDPG algorithm to support the DQN algorithm with a continuous trajectory to achieve better energy efficiency and a better UAV trajectory in the system. Therefore, their DDPG-based solutions gained higher rewards than their DQN-based solutions did, as DQN only tried a limited set of actions, while DDPG continuously optimized the variables. However, the system design with the implementation of DDPG had a higher computational complexity due to its more complicated architecture, which implemented two DNNs as an actor network and a critical network. It also resulted in the training time required by DDPG being longer than that required by other implemented algorithms.

The authors of [60] leveraged the DDPG algorithm to overcome the loss of accuracy gained by formulating continuous variables into discrete variables in the DDQN algorithm. The computational complexity of systems with DDPG was similar to those with DDQN because both used double DNNs. However, the characteristic of DDPG being able to handle continuous actions resulted in a decrease in propulsion energy of up to 10.52% and an increase in energy efficiency of up to 31.55% compared to DDQN. The results of the 3D trajectory and its projection in the 2D plane showed that the communication quality received by users in DRL-algorithm-based systems was better due to the UAV’s tendency to approach the RIS to find suitable paths for serving users.

In [62], Huang et al. implemented the DDPG algorithm to maximize the sum rate capacity of RIS-assisted multiuser multiple-input–multiple-output (MIMO) systems. In that study, a DNN was used to map the channel state information (CSI) as the primary parameter value sent by the environment. The matrix values of phase shift and beamforming were optimized by considering the given CSI. Based on (5), the state value was the input for the actor and critic network. However, the correlation between states as inputs could reduce the value of the NN efficiency as an approximation function. Therefore, the state entered a whitening process to remove its correlation before inputting both networks. In addition, batch normalization was utilized in the hidden layers to overcome the variations in the distribution of each layer’s input resulting from the changes in the parameters of the previous layers. Using these solutions, the DDPG algorithm in this study was able to provide optimal beamforming and RIS phase shift and to produce comparable sum rate performance with that of the state-of-the-art benchmarks with the cost of the system’s complexity. However, the primary purpose of implementing the DDPG algorithm in this study was to obtain the optimal beamforming and RIS phase shift, rather than to train an NN for online processing.

The DDPG algorithm can also be implemented to optimize the RIS phase shift by considering the CSI in a high-speed railway network [63]. To avoid the performance loss caused by phase-shift design with an outdated CSI, some authors calculated the delay value between the outdated CSI and real-time CSI. The result illustrated that the performance loss grew by up to 15.1% with the decrease in the outdated CSI coefficient. That was because the lower value of the outdated CSI coefficient made the CSI more inaccurate, which added to the difficulty of optimizing the RIS phase shift. In addition, in this study, an RIS was deployed near a mobile relay to suppress the interference signal. As the number of RIS REs increased, the result showed that the proposed system produced a capacity that was up to 212% higher than that of a system with a random phase shift and was significantly higher than that of a system without an RIS. Thus, it was explained that increasing the capacity without optimizing the REs was inefficient.

Ma et al. in [64] implemented DDPG for RIS-aided multiuser MISO systems with hardware impairments to maximize the user data rate by optimizing the beamforming and phase shift of an RIS. This study observed the minimum average user data rate, where the value increased by up to 22.22% as the RIS REs increased. However, the result also illustrated that the increase in REs did not affect the convergence speed of the proposed DDPG algorithm. The proposed system was also robust in a uniform distribution of channel communication. By comparing the performance achieved with that of the non-optimized algorithm, the proposed system significantly outperformed the existing non-optimized algorithm by up to 125%. However, to reduce the proposed system’s computational complexity and feedback overhead, they considered the design of the transmission scheme based on statistical CSI because the beamforming and RIS phase shift needed to be calculated in channel coherence intervals for instantaneous CSI-based schemes, which increased the computational complexity.

Other than that, the implementation of DDPG to optimize an RIS-based UAV-NOMA downlink network was considered in [65]. The authors initiated the previous user’s data rate, the angle of RIS phase shift, and the UAV’s horizontal position to obtain the new RIS phase shift for maximizing the downlink users’ sum rate. DDPG was implemented to ensure the successful implementation of successive interference cancellation instead of conventional optimization methods, such as convex optimization, which requires much mathematical processing. The achieved result showed that the proposed system significantly outperformed the system with a random phase shift as the transmit power and number of REs increased. Even though the proposed system was always convergent and stable with various numbers of REs, it was essential to consider the tradeoff among the number of users, the number of REs, and the data rate. The increase in the number of RIS elements was directly proportional to the number of neurons and the duration of training required, where a longer training duration and a greater number of neurons led to a higher calculation complexity and made the output latency non-negligible.

The authors of [66] considered the transmitter channel, previous phase, and previous estimated SINR provided by the environment for the agent to obtain new RIS phases in order to maximize the sum rate of an IRS-assisted NOMA system. The authors implemented another algorithm, the exhaustive search algorithm, to compare the performance of the proposed system. The result revealed that the NOMA sum rate generated by the DDPG algorithm approached the upper bound and was close to optimal. The proposed system achieved a sum rate that was 6.56% higher than that of the exhaustive search algorithm. In addition, the proposed system also increased the sum rate by up to 12.5% as the number of users increased. In addition, the proposed system’s computational complexity was much lower than that of the exhaustive search algorithm due to the considered parameters.

**Table 3:** Summary of the DDPG algorithms for RIS optimization.

References	Problem	Optimized Parameters	Implemented RL Algorithm	RIS Installation
[59]	Maximizing the energy efficiency of a UAV	1. UAV trajectory 2. RIS phase shift	DQN and DDPG	Attached to a building
[60]	Maximizing the data rate and reducing the loss of accuracy	1. Continuous UAV trajectory 2. Continuous GT scheduling	DDQN and DDPG	Aerial RIS
[62]	Maximizing sum rate capacity	1. Transmit beamforming 2. RIS phase shift	DDPG	Attached to a building
[63]	Maximizing the capacity with interference	1. RIS phase shift	DDPG	Attached to a moving vehicle
[64]	Maximizing the user’s data rate	1. Transmit beamforming 2. RIS phase shift	DDPG	On the ground
[65]	Maximizing the downlink user’s data rate	1. BS power allocation 2. RIS phase shift 3. UAV horizontal position	DDPG	Aerial RIS
[66]	Maximizing the long-term average of users	1. RIS phase shift	DDPG	On the ground
[67]	Maximizing the sum secrecy rate	1. UAV active and passive beamforming 2. RIS reflecting beamforming	TDDRL	Attached to a building

Apart from the previous studies, in [67], Guo et al. aimed to maximize the sum secrecy rate by implementing twin-DDPG deep reinforcement learning (TDDRL) for RIS-aided millimeter-wave UAV communications. The first DDPG was responsible for learning the optimal policy of the UAV beamforming matrix and RIS-reflecting beamforming matrix. Another DDPG network was responsible for obtaining the optimal movement of UAV beamforming and RIS-reflecting beamforming. Similarly to [63], the authors considered the delay between the outdated CSI and real-time CSI to avoid performance degradation. The computational complexity of the TDDRL algorithm depended on the number of DNN layers exploited in each DDPG network, which could be reduced by cutting training procedures after the network performance converged. The result illustrated a gap between the average sum secrecy rate achieved by the proposed system and that of the single DDPG because the proposed system had more potential to separate complicated variables, such as the CSI and UAV positions.

For a DDPG-based system, the complexity of a trained network depends mainly on the actors and the network architecture. Although there have been plenty of studies that have worked with DDPG to overcome the drawbacks of DQN in terms of the continuous action space, DDPG has a laxity considering that all of the descendants of Q-learning suffer from overestimation bias.

### 3.3. Twin Delayed DDPG (TD3)

Similar to the DDPG algorithm, TD3 is a policy-based RL method and an actor–critic algorithm. However, TD3 could reduce the overestimation bias obtained from the descendant Q-learning by implementing two critics: $Q_{\phi_{1}}^{{}_{\pi_{\theta }}}$ and $Q_{\phi_{2}}^{{}_{\pi_{\theta }}}$. TD3 computed the target as the minimum value between two critics, as shown in (8), where DDPG calculated the target value based on the maximum Q-value.

(8) $$y=r+\gamma \underset{i=1,2}{min}Q_{\phi_{i}^{\prime }}^{}\left(s^{\prime },\tilde{a}\right)$$

where *ã* is the action obtained with the addition of a small random noise.

TD3 can be implemented with variance-lowering optimization, which includes delayed policy updates and target policy smoothing. Delayed policy updates are where one actor and target are updated for every two critics in order to obtain a higher quality of the target value. Target policy smoothing is adding random noise to the target action chosen by the deterministic policy at each training step in order to keep the target action close to the actual action. The TD3 algorithm trains the actor by using a deterministic policy gradient over the action to update the actor’s weight, and one of the two critics $\phi_{1}$ is trained with the action. The deterministic policy gradient for TD3 is shown in (9).

(9) $$\frac{J\theta }{\partial x}=\frac{1}{N)}\sum_{i}^{}\frac{Q_{\phi_{1}}^{{}_{\pi_{\theta }}}\left(s,a\right)}{da}\frac{\pi (s|\theta )}{d\theta }$$

Several existing studies discussed in this subsection regarding the implementation of the TD3 algorithm for RIS technology are briefly summarized in Table 4.

In [68], Hashemi et al. implemented the TD3 algorithm to maximize the total achievable finite block length rate in all actuators for short packet communication in RIS-assisted networks. The TD3 algorithm optimized the RIS phase-shift matrix by considering the channel’s response to the local information. The authors used DDPG for a comparison of the system’s performance. The result illustrated that the proposed system had fewer fluctuations in the average finite block length rate than the DDPG did. Due to the more stable fluctuations, the learning speed in phase control became faster. The system also captured the Shannon rate and finite block length rate to analyze the system’s performance in ideal or non-ideal reflective phase-shift design. The result showed that the Shannon rate increased by up to 7.7% and the finite block length rate increased by up to 28.57% when implementing an ideal RIS. In addition, the proposed system was proven to be practical in ideal or non-ideal RISs because the graphs’ slopes were quite similar as the number of REs increased.

In [69], the authors implemented TD3 for RIS-assisted multi-antenna ambient backscatter communication (AmBC) signal detection. The RIS controller, as the agent, received the CSI from the AmBC system to optimize the RIS phase shift and obtained the maximum energy ratio of the systems. The authors used another DRL algorithm (DDPG) and conventional algorithms (successive convex appropriation and semi-define relaxation) for performance comparisons of the proposed systems. The simulations proved that the proposed system had an increase in performance quality of up to 23.8% with a cost of the time consumed being 0.03 s longer and the application of two more DNNs than in DDPG. Meanwhile, the conventional algorithm had the lowest-quality performance and consumed 1.3 times more time than the proposed system did, proving that the proposed system had a significantly lower complexity than that of the conventional algorithm. In addition, the achieved result also showed the effect of the hidden layer on system performance; with an increase in implemented hidden layer, the system performance gradually decreased due to the DNN becoming more extensive, which resulted in increased learning and training complexity.

TD3 could also be implemented for joint optimization of the RIS phase shift and precoding matrix [70]. Some authors aimed to obtain the maximum value of the sum rate by running a policy evaluation at the end of each learning episode by considering the channel responses from the users to the RIS and from the RIS to the BS. The proposed system was compared to a model-drive minimum mean square error based on an alternating projected gradient algorithm. It showed a better sum rate that outperformed the baseline in low inter-user configurations. In addition, the performance quality of the proposed system increased by up to 72.73% with respect to another algorithm when simulated in conditions with a high SNR.

The aforementioned studies showed that the TD3 algorithm can be a solution for improving system performance compared to that of other Q-learning and conventional algorithms. However, even though the TD3 algorithm has levels of computational complexity and time consumption that are worth with its communication performance, it is essential to consider the tradeoff among the number of users, the number of REs, and the number of hidden DNN layers. However, there are still very few studies that have applied the TD3 algorithm to RIS-assisted systems.

**Table 4:** Summary of the TD3 algorithm for RIS optimization.

References	Problem	Optimized Parameters	Implemented RL Algorithm	RIS Installation
[68]	Maximizing the total achievable finite block length rate	1. RIS phase shift	TD3	On the ground
[69]	Maximizing the energy ratio	1. RIS phase shift	TD3	On the ground
[70]	Maximizing the sum rate	1. RIS phase shift 2. Precoding at transmitter	TD3	On top of building

### 3.4. Proximal Policy Optimization (PPO)

Unlike the previous algorithms based on Q-learning, the PPO algorithm is based on a ratio between the current policy $\pi_{\theta }\left(s_{t}|a_{t}\right)$ that will be learned and the baseline policy $\pi_{\theta_{k}}\left(s_{t}|a_{t}\right)$ that was obtained from previous experiences. The ratio between these policies is notated as $R_{t}\left(\theta \right)$ and is shown in (10).

(10) $$R_{t}\left(\theta \right)=\frac{\pi_{\theta }\left(s_{t}|a_{t}\right)}{\pi_{\theta_{k}}\left(s_{t}|a_{t}\right)}$$

However, if the probability ratio between the new and old policies is outside the range of (1−ε) to (1+ε), the advantage function will be clipped by using a clipped objective function, as shown in (11).

(11) $$L_{\theta_{k}}^{CLIP}\left(\theta \right)=\underset{\tau ∼\pi_{k}}{E}\left[\sum_{t=0}^{T}min\left(r_{t}\left(\theta \right){\hat{A}}_{t}^{\pi_{k}},g\left(\epsilon ,{\hat{A}}_{t}^{\pi_{k}}\right)\right)\right]$$

where

(12) $$g\left(\epsilon ,A\right)=\left\{\begin{array}{ccc} (1+\epsilon )A & A\geq 0 & \\ (1-\epsilon )A & A<0 \end{array}\right.$$

where the advantage function $A^{\pi }\left(s,a\right)$ and value function $V^{\pi }\left(s\right)$ are, respectively, the parameter for knowing how much an actor is better than expected and the parameter for measuring how good the current state is, as shown in (13) and (14).

(13) $$A^{\pi }\left(s,a\right)=Q^{\pi }\left(s,a\right)-V^{\pi }\left(s\right)$$

(14) $$V^{\pi }\left(s\right)=E\left\{\sum_{t\geq 0}^{}\gamma^{t}r_{t}|S_{0}=s,\pi \right\}$$

In [71], Nguyen et al. aimed to maximize the energy efficiency for all users in RIS-assisted multi-UAV networks. Two agents were implemented. The UAV agents optimized the UAV power allocation, and the RIS agents optimized the phase-shift matrix with the given channel gain to maximize the system’s energy efficiency. The authors proposed two system schemes that used the PPO algorithm: a centralized scheme and a parallel learning scheme. The difference between the two was the training starting time for the policy to maximize the EE performance. The policy was used and trained in the centralized scheme when the system had an N+1 policy for UAV N and policy N for the parallel learning scheme. The converged parallel learning PPO scheme was faster than the other schemes. In addition, the energy required for the proposed parallel system was 92.47% more efficient, and the centralized PPO system was 26.67% more efficient compared to systems with random phase shift schemes as the number of users increased. Meanwhile, along with the increase in RIS REs, PPO parallel learning was 3.4% more efficient than the centralized PPO algorithm.

Another study aimed to minimize the information age by optimizing the UAV altitude, the communication schedule, and the RIS phase shift [72]. The proposed PPO algorithm did not rely on prior knowledge of the activation patterns. It found the control policy controlling the UAV altitude and the scheduling decision within an unknown activation pattern by considering the SNR and RIS phase shift of the previous action. The authors used the UAV altitude and scheduling policy as the main control objectives to reduce the DRL learning complexity and as a tradeoff due to the discretization of the altitude of the UAV and phases of the RIS elements into discrete actions. In addition, the proposed PPO architecture was also constructed with the same number of neural units in all hidden layers to reduce the computational complexity. The results showed that as the number of users increased, the expected sum of the age of information of the proposed system decreased by up to 57.7% with respect to the other two basic policies. The result also showed that increasing users’ transmit power and the number of RIS REs could enhance the SNR achieved at the BS. However, an increasing number of transmit power may not be allowable in certain IoT applications due to the decrease in energy efficiency. Therefore, increasing the number of RIS REs is another solution for enhancing communication quality and simultaneously increasing the SNR and expected sum of the age of information.

The authors of [73] implemented PPO to empower passive beamforming and routing design for multi-RIS-assisted multihop networks. The PPO algorithm considered the route node, available power allocation, and channel capacity to optimize the route node and power allocation for the next transmission with the aim of maximizing the minimum end-to-end data rate among routers. The simulation results showed that the proposed system with the PPO algorithm produced a data rate that was 33.7% higher than that with a random RIS coefficient and 34.3% higher than the system without an RIS. However, it was unavoidable that as the number of RIS REs increased, the percentage increase in the data rate decreased. In addition, the power allocation also impacted the data rate produced by the system. The data rates in systems that allocate power to weak links increased by up to 30.46% with respect to the data rates in systems without power allocation. The studies mentioned in this subsection show that the PPO algorithm can improve the performance of RIS-assisted systems. However, even though the PPO algorithm is able to improve system performance—whether single- or multiple-RIS conditions—consideration of the number of users, the number of REs, and the allocation of power is essential for optimizing the energy efficiency obtained by the system.

An in-depth study explained the importance of the number of REs, since this is the main parameter in an RIS. Maximizing the communication quality and energy efficiency with the minimal number of REs can be a future approach in this field, as it can enhance the system’s performance. Table 5 provides a brief summary of papers discussed in this subsection.

## 4. Potential Challenges and Future Research Opportunities

Based on the explanation in the previous section, some studies have implemented RL for RIS technologies. However, future research must consider several issues, which will be discussed in this section, to improve systems’ performance even further.

### 4.1. Optimal RIS Placement

RIS placement is an important factor that needs to be considered because RISs are widely applied to overcome signal transmission problems in areas with blockages and to transmit signals by minimizing distortion and interference. Therefore, the placement of an RIS can determine the efficiency of the reflected signal and the optimal level of the distributed signal with a high optimal achievable rate [74,75,76]. In addition, the size of an RIS and the number of REs can also affect a system’s performance. The size of an RIS and the number of REs can increase the percentage rate of signal reflection in the right direction at the cost of high overhead [77,78].

### 4.2. Channel Estimation

Channel response is considered the main parameter for optimizing the RIS parameters because a channel includes all kinds of parameters that can affect a system’s performance, such as fading, scattering, and shadowing. Accurate CSI is essential in RIS-aided wireless communications [79]. However, in actual implementations, it is a challenge for RIS-assisted wireless networks to continuously achieve accurate CSI values due to the flexibility of the clients served and the signal’s character of being prone to obstacles. As a result, the problem of identifying the CSI and optimizing the network performance with a poor CSI must be appropriately addressed to enable real-time and effective RIS-assisted transmission. The RL approach can be one of the solutions for overcoming these problems. However, implementations with RL require a longer time due to the need for the system to carry out data training with constantly changing channel conditions. Thus, further research is needed to investigate accurate channel estimation without increasing training overhead, which will also impact the power consumption.

### 4.3. Power Consumption

RISs are developing technologies for overcoming the main weaknesses of conventional relays, and they can forward signals as passive or active relays [80]. However, adding power to an RIS, which makes it work as an active relay, will increase the amplitude of the signal, which may lead to an increase in the communication capacity and a decrease in the bit error rate [81]. However, the efficiency and battery capacity level must be considered when implementing an active RIS [82]. Another scenario is when an RIS is carried by a mobile technology with a limited power supply, such as a UAV. In these circumstances, power consumption is a crucial concern that needs to be addressed. Both ML and RL can be applied to perform resource management and power allocation in the system; these are solutions that can allow problems related to power consumption and improve energy efficiency to be overcome. However, paying attention to the model training time that ML and RL must use is necessary. Therefore, further research on achieving energy efficiency without high overhead is needed.

### 4.4. Model Training

In DRL, iteration for training and testing is the primary concern because it requires a long time. The longer it takes to perform the iterations, the more likely it is to obtain the optimal parameter value [83]. An optimization function can be a possible solution for minimizing the training duration in DRL. There are several of them that can be adapted to such systems, such as the mini-batch gradient descent, RPop, RMSProp [84], Adagrad [85], Adam [86,87], etc.

### 4.5. Federated and Split Learning for RISs

Federated learning (FL) is part of decentralized ML, where the central node (server) broadcasts the entire model to each user (client). This learning method is suitable for networks consisting of central nodes and several clients, such as RIS-based networks. The way in which federated learning works, which allows clients to use individual training models, means that users do not have to share their data with the server. Thus, the implementation of FL will allow an increase in the secrecy rate of the system to be overcome. In [88], FL was applied to optimize power allocation and resource scheduling in UAV-assisted networks. Thus, FL can also be implemented in RIS communication in the same context. Another learning method that can be a solution to problems related to secrecy performance, power allocation, and spectral efficiency is split learning (SL). SL has a way of working that is similar to that of FL. However, in SL, each client is only responsible for each layer, so there is no need to train the entire model. Therefore, the implementation of SL can allow the high overhead issue due to the number of iterations required to be overcome. Further in-depth research is needed to prove the capabilities of both of these learning methods.

### 4.6. RISs for Underwater Wireless Communication

The implementation of the RIS-assisted Internet of Underwater Things (IoUwT) has yet to be reached by many researchers. In underwater communication, signals are sent in optical or EM waves. However, the characteristics of both of these types of waves only allow short-distance communication to occur [89]. Another problem in underwater wireless communication is the high level of multipath fading caused by water flow [90], the movement of underwater living things, and uneven surfaces, which can reduce the data rate. RIS technologies can be a solution to these problems by minimizing the multipath effect and enhancing the data rate. The implementation of RIS technology in an underwater medium can be achieved by placing it on the seabed, allowing it to float below the sea surface, or carrying it with an autonomous underwater vehicle or an autonomous surface vehicle. An in-depth research effort regarding the implementation of RISs for IoUwT needs to be carried out in the future.

### 4.7. RISs for Underground Wireless Communication

In order to achieve excellent wireless communication anywhere, underground media should be further investigated by researchers. Wireless communication in an underground medium with high reliability and high data rates is increasingly needed along with the development of the Internet of Underground Things (IoUgT) in mines and tunnels for applications in various sectors, such as agriculture, earthquake mapping, underground boundary protection, rescue, learning needs in geology and geography, etc. However, obstacles in underground media, such as rockfalls and mining equipment, can cause ray-path blocking and are a concern that needs to be addressed [91]. RISs are able to overcome these obstacles by reflecting a signal in the desired direction, so enhancing the signal quality even increases its data rate. Multipath fading effects, such as scattering and reflection due to tunnel walls, as well as the natural movement of the soil with different absorption levels, are unavoidable problems, and they result in uncontrolled signal reflection directions. RL’s ability to make the best decisions in adaptive situations can be a solution for overcoming these intractable problems. With an applied fixed-position sensor, an underground map can be used as a benchmark for estimating the ideal RIS placement.

## 5. Conclusions

RIS technology can be implemented in several types of dynamic and static communication systems. The option of placing an RIS, which can be on the ground, attached to a wall, on top of a building, or suspended in the air, will benefit systems because it can transmit signals precisely to a user’s position by avoiding distortion and interference between the transmitter and receiver. The precise direction of the signal will provide other benefits, such as enhancing the data transmission, maximizing the sum rate and energy efficiency, and minimizing the information age. In addition, the DRL algorithm, which allows a machine to automatically make decisions based on experience, will make it easier for systems to optimize signals sent by RISs. This survey described an overview of RIS technologies and the application of RL—especially DRL—for RIS technologies, which are promising for future wireless communications. RISs can overcome the significant drawback of conventional relays related to previous technologies, as RISs can transmit signals actively or passively and almost without needing any additional power. In this article, we focused on the implementation of DRL to optimize RIS parameters, such as optimization for passive beamforming, phase shift, RIS placement, etc. However, even though the application of RL in IRSs showed good potential in previous studies, several things still need to be considered for further research. In order to obtain signals with a high sum rate, minimal information age, and high energy efficiency, it is essential to think carefully about the location of IRS implementation and the implemented algorithm. Various insights into and possible solutions for several open challenges that can be discussed in the future are provided at the end of this study, such as the importance of optimization for channel estimation, RIS location, energy and cost efficiency, data and model training for DRLs, and other areas of wireless communication that can be assisted by RIS technologies with the DRL algorithm.

## Figures and Tables

**Table 1:** List of works surveyed on the implementation of RL for RISs.

References	Year	Thoroughly Explained Scope of the Architecture					Limitations and Contributions
		General Knowledge of RL	DQN	DDPG	TD3	PPO	
[21]	2021	✔	x	x	x	x	RIS deployment and system design
[22]	2021	✔	x	x	x	x	RIS hardware and system design
[23]	2022	✔	x	x	x	x	IRS-assisted UAV for massive networks in ground and airborne scenarios
[24]	2022	✔	x	x	x	x	IRS-assisted UAV for non-terrestrial networks
[25]	2022	✔	x	x	x	x	Optimization and performance analysis for UAV-assisted RIS communication
[26]	2022	✔	x	x	x	x	Channel estimation and RIS based on ML applications
[27]	2022	✔	x	x	x	x	Signal processing and AI methods for RIS phase-shift optimization
Our work	2023	✔	✔	✔	✔	✔	RL algorithms implementation for RISs

**Table 2:** Summary of the DQN algorithms for RIS optimization.

References	Problem	Optimized Parameters	Implemented RL Algorithm	RIS Installation
[59]	Maximizing the energy efficiency of the UAV	1. UAV trajectory 2. RIS phase shift	DQN and DDPG	Attached to a building
[60]	Maximizing the data rate	1. UAV trajectory 2. RIS passive phase shift 3. GT scheduling	DDQN and DDPG	Aerial RIS
[61]	Mitigating over-estimation and maximizing average sum rate	1. RIS passive phase shift	DDQN	On the ground

**Table 5:** Summary of the PPO algorithm for RIS optimization.

References	Problem	Optimized Parameters	Implemented RL Algorithm	RIS Installation
[71]	Maximizing the energy efficiency	1. UAV power allocation 2. RIS phase shift	PPO	Attached to a building
[72]	Minimizing the information age	1. UAV altitude 2. Communication schedule 3. RIS phase shift	PPO	Aerial RIS
[73]	Maximizing the minimum end-to-end data rate	1. RIS phase shift 2. User’s power allocation 3. Next transmission route node	PPO	On the ground

## Data Availability

Not applicable.

## Abbreviations

The following abbreviations are used in this manuscript:

Adagrad	Adaptive Gradient
AI	Artificial Intelligence
AmBC	Ambient Backscatter Communication
BS	Base Station
CSI	Channel State Information
DC	Direct Current
DDPG	Deep Deterministic Policy Gradient
DNN	Deep Neural Networks
DDQN	Double Deep Q-Network
DQN	Deep Q-Network
DRL	Deep Reinforcement Learning
EM	Electromagnetic
FL	Federated Learning
GT	Ground Terminal
IoUgT	Internet of Underground Things
IoUwT	Internet of Underwater Things
IRS	Intelligent Reflecting Surface
LoS	Line of Sight
MDP	Markov Decision Process
MIMO	Multiple Input Multiple Output
ML	Machine Learning
NLoS	Non-Line of Sight
NOMA	Non-Orthogonal Multiple Access
PPO	Proximal Policy Optimization
RE	Reflecting Elements
RF	Radio Frequency
RIS	Reconfigurable Intelligent Surface
RL	Reinforcement Learning
RMSprop	Root Mean Square Propagation
RProp	Resilient Propagation
SGD	Scholastic Gradient Descent
SL	Split Learning
SNR	Signal-to-Noise Ratio
TD3	Twin Delayed DDPG
TDDRL	Twin-DDPG Deep Reinforcement Learning
UAV	Unmanned Aerial Vehicle
UWOC	Underwater Wireless Optical Communication
